# French fashion and textile during COVID-19. Once again, flying to high quality and innovation to survive

**DOI:** 10.1186/s40691-021-00278-1

**Published:** 2021-12-15

**Authors:** Bertrand Blancheton

**Affiliations:** Professor, GREThA UMR CNRS 5113, Dean of Economic and Business Faculty, Faculty of Economy and Business, University of Bordeaux, Avenue Duguit, 33 608 Pessac, France

**Keywords:** Textile industry, France, Covid-19, Luxury, Quality, Innovation, L15

## Abstract

This article studies the consequences of the COVID-19 global health crisis on the fashion and textile industry in France. This crisis is only part of an already long, slow decline in the industry. The paper analyzes the composition and organization of textile industry. It offers data related to: sales, consumption, employees, company size, as well as imports and exports - all highlighting the importance of fashion and textiles in France today. The paper shows how lockdown has asphyxiated production and retail sales. COVID-19 caused the appearance of new challenges: mask production, new aspects of CSR in luxury textiles and the development of antiviral fabrics. Mask production can be considered as a case study useful in the analysis of textile challenges. In this context, the French textile industry should continue to improve on innovation and quality. Promoting labelling on the global market can help the sector to develop its high-end. France is credible to expanding its fashion and textile supply in luxury.

## Introduction

The goal of this paper is to analyze the consequences of COVID-19 on French fashion and textile industry. The COVID-19 disease is the archetype of an exogenous shock on economics. Even if economic history shows that exogenous shocks are relatively frequent (meteorology, energies, pandemics) COVID-19 is truly unique and cannot be compared to the rest. The shock has been symmetrical and particularly violent when it comes to political health protection choices. Strict lockdowns affect all sectors to different magnitudes. In France, the GDP fell 8% in 2020 and unemployment sky-rocketed.

In France, COVID-19 has given credibility to and exacerbated the debate on de-globalization. National independence may motivate turning to possibilities like pushing some strategic sectors into reshoring. The crisis has also obligated the textile value chain to restructure and revealed new social responsibility aspects in the fashion industry. Luxury groups have granted financial aid to hospitals and have launched clothing workshops in order to assist in the production of masks and gowns. Some have even made masks a fashion item, others have refused. The crisis has been shedding light on the industry’s capacity to innovate: antiviral fabrics for example.

In May 2020, French textile production plummeted to levels as low as 70 to 80% below regular production. Several big retail brands are experiencing considerable difficulties (Célio, Camaieu, Damart). This article demonstrates that in the French textile industry context, innovation should continue to be promoted and quality increased in line with the new international economic model (Krugman, 1979). Empirical literature is evidence for innovation’s crucial influence on the industry’s ability to survive (Ju & Lee, 2020; Lang et al., 2016; Nayak & Mishra, 2016; Sokolowski, 2020; Villa & Kuster-Boluda, 2007). This paper explores different issues in order to improve French textile quality and competitiveness on the global scale: high-end and luxury advances founded on CSR, labelling and cluster benefits.

According to the goal of the paper which is to analyze the consequences of COVID-19 on French fashion and textile industry during a lockdown we decide to propose a monograph. Qualitative approach founded on interviews are so complex to drive in this special context. This paper is a monograph put together using historical, economic and business academic evidence. Corporate and union websites, national press sources like “La Tribune”, “Les Echos”, “Le Figaro”, “Le Monde” and specialized press like “L’Usine Nouvelle” and “Vogue” have been used extensively to complement the written sources. This article also offers data that highlights the importance of fashion and textiles in France. We assemble data sources on textiles in international trade (“Tableau général du commerce de la France”) and in national production (INSEE and OPCALIA). We use these sources to characterize the textile industry in terms of sales, consumption, employees, company size, imports and exports. We highlight current organization of French textile production and debates on its future evolution. We try to show the impact of the COVID pandemic on the textile industry and company activities.

This article is structured as follows. Section "Case description" presents case description in particular an historical perspective of textile industry in France up to COVID-19 and today’s current organization of national production. It shows how lockdown has asphyxiated production and retail. It focuses on mask issues as example of CSR. Section "Discussion and evaluation" proposes a discussion on quality-based strategy that promotes survival on today’s modern global market. Finally we give conclusions.

## Case description

### Historical perspective

Literature on French textile industry highlight a long decline and the low weight of the sector in national industry. For a couple of years now, the textile sector in France has shown its survivor instinct, an observation that can also be said for Spain (Santos-Roldán et al., 2020). However in truth, the French textile industry has actually been experiencing a long and slow decline since the ninetieth century. This new crisis has only served to further accelerate that decline.

Textiles have long been a strong French specialization. From 1835 to 1844, textiles (including clothing and fabric works) represented 42% of industry added value. Silk, wool and cotton fabrics with varying quality levels, were the principal exports in French international trade (Becuwe & Blancheton, 2020). From the mid-ninetieth century, clothing emerged as a new area of French expertise made possible by Paris’ global reach (Becuwe et al., 2018). In 1858 Charles Frederick Worth created the first true fashion house in Paris. In the years to come, Gustave Beer (1886), Callot Soeurs (1895), Coco Chanel (1906), Madeleine Vionnet (1912) and Jean Patou (1912) would follow suite, creating famous brands and building Paris’ reputation as a fashion capital. Clothing export unit values increased significantly. Shortly after WWI, silk fabrics enjoyed a short stint as the number one French export, representing 12% of total exports in 1919 (Tableau Général du commerce de la France). However, from 1935 to 1938, the textile industry’s added value dropped to 23% (Becuwe & Blancheton, 2020). Christian Dior (1946) and Yves Saint-Laurent (1961) amongst other designers, brought new life to French fashion and further reinforced Paris’ status as a fashion capital of the world. However, not even their positive influences were able to counteract the clear decline of the textile industry in France.

The second phase of modern globalization was fatal to the French textile industry as it was unable to compete with emerging foreign market prices. At the end of the 1980s, French producers were already noncompetitive in capital-intensive activities and low-cost products (Battiau, 1991). The ratio of investment to sales was not high enough, and it was difficult for French manufacturers to adapt to new trends appearing in global clothing markets. By the 1990s, a large gap in income had started to surface between the knitted fabrics industry in competition with emerging markets and the manufacturing of high-end clothing which was prospering, particularly wool combing and sewing-thread manufacturing. Innovation and quality became the only ways to survive during this phase.

In 2018, according to INSEE, textiles represent only 2% of added value in the manufacturing industry. Over the last twenty-five years, it has lost two thirds of its workforce and more than half of its production. France is now massively importing "textile" products, especially clothes and shoes, half of which come from Asia and a third from Europe. Since 2008, fashion and textile consumption has fallen from year to year (except 2017 (+0.6%)). Recorded sales were only € 29.3 billion in 2018.

French manufacturers can be found all across the country, though certain regions have richer histories than others and are therefore more deeply rooted and woven into the regional industry fabric (Normandie, Vosges, Drôme, Rhône, Nord…). Combining the efforts of these manufacturing regions does not yield significant positive results (Hlady-Rispal & Blancheton, 2020) however each region has its own distinctive value proposition based on its local heritage.

### Composition and organization of textile industry in France

According to the French National Statistics Organization (INSEE), the value of textile production amounted to € 16.4 billion in 2015. Five categories of products made up two-thirds of production: clothing (19%), leather goods (18%), made-up textile articles excluding clothing (10%), "technical and industrial textiles" (9%) and fabrics (8%). Traditional products, typical of high-end expertise such as leather goods, rub shoulders with the “technical and industrial textiles” category which consists of high-tech industrial products such as reinforced and ultra-resistant textiles used in the aeronautical and automotive industries. According to the French Textile Industry Union website “l’Union des Industries Textiles”, France’s textile industry was made up of 2164 companies in 2018, employing 61,296 people with an annual turnover of approximately € 13.6 billion.

French production is mainly organized around large companies of 200 or more employees, especially multinationals (Table 1). These groups specialize in the production of luxury items or textiles with high added value such as technical textiles used in the aeronautical and automotive industries (examples: Petit-Bateau in Troyes or Aigle International in Vienne). In regard to clothing the little industrial activity remaining in France is mainly that of industrial contractors and foreign subcontractors. In 2018 French textile exports represented € 9.4 billion (exports of goods € 491 billion) and imports € 16.9 billion (imports of goods € 551 billion). China remains the main source of textile imports for France followed by Bangladesh and Italy (Blancheton & Chhorn, 2019).

**Table 1:** Distribution of employees according to company size in the French textile industry in 2017

Number of workers	Share of companies in total (%)
[0–10]	8
[10–50]	26
[50–200]	29
[200–499]	22
[500–1999]	15

Sources: Opcalia TMC. Opcalia is an association which collected contribution of companies for training in different professional branches including textile

The COVID-19 crisis has started a debate on de-globalization and reshoring France’s strategic sectors. In France, politicians mourn the days of national dependence in certain sectors (health, chemistry, etc.). The definition on what constitutes a strategic sector depends largely on context. Today, defense activities, energy, health, food, big data and communication are considered crucial. An economy must be evenly diversified in order to be able to absorb shocks; this is achieved by rapidly reorganizing production to keep it stable. Sectors must also be intelligible and flexible. Textile is part of both a traditional industry and the old economy. On this basis, relocating textile production back onto French soil cannot be considered as a realistic option. An international labor force and a complex value chain (Bruce et al., 2004) are not compatible with a textile that is fully made-in-France. A company approach with this mission (CSR 100% made-in-France) just seems to be a niche strategy like “le Slip Français” (men’s underwear) case. Fashion needs extensive product ranges and low-cost stock in order to satisfy local demand.

Reshoring is not a panacea in terms of national job creation (Mouhoud El Mouhoub, 2017) and activity. Mouhoud El Mouhoub shows that industrial reshoring is generally capital intensive (robots, machines). Reshoring cannot be considered as a realistic solution to fight unemployment in France.

### An asphyxiated sector during the COVID-19 global health crisis

Due to the government decision, during the strict lockdown in France (March 17th to May 11th, 2020), French textile production was almost completely stopped. By May production levels had fallen to as low as 20–30% of normal figures. Textile companies that supply the tourism sector, aeronautical and automotive industries have been severely impacted with the only continuing production being that for healthcare. According to the president of “l’Union des industries textiles” Yves Dubief, “to tell the truth, the sector is only surviving thanks to the fabrication of masks”. Companies’ motivations were ethical and economic. In 2 months, the textile industry has gone from zero to between 4 and 5 million units produced per day. Far from stopping, this production must be maintained until long after the end of the crisis. Also, according to Yves Dubief, throughout France, the manufacturing of masks safeguards more than 10,000 jobs. Proximity delivery times are strictly adhered to. French companies use recyclable fabric and guarantee sanitary tracking as well as a price per unit (average production cost between € 0.15–0.20). In June, the French administration continues to order masks and an overproduction is rapidly appearing. A sizable amount of the population prefers low cost masks from China.

According to INSEE textile consumption, French textile sales have tumbled to as low as 15% over the last 10 years. A secondary market on-line has been established in fashion (Vinted, Le bon coin, etc.). In retail, market structure is very atomistic and brands are fragile. In accordance with industry experts, postponed summer sales (from July 15th to August 11th) however were still not successful.

COVID-19 is accelerating the difficulties of several big names in entry-level fashion, such as Célio, Naf Naf, Chipie, Catimini, Kenzo Kid, La Halle or Camaïeu. In 2020, national press describes regularly that employment in the sector is about to pay a heavy price.

- In June 2020, Celio, leader of the male market in France, asked the commercial court of Bobigny to be placed on the bankruptcy watchlist. The chain founded in 1978 by Laurent and Marc Grosman, employs more than 4000 people worldwide and has 488 shops in France. They explained that like other competitors, they are facing a severe cash crisis and therefore can’t get credit.
- Placed in receivership, Naf Naf was recently taken over by its supplier, Sy, a Turkish group, in June, which has retained 75% of its employees. The Bobigny Commercial Court ordered the Naf Naf bankruptcy, 2 years after its acquisition by a consortium of investors led by Chinese group La Chapelle, in Vivarte. This group has been engaged for several years in an asset disposal and restructuring plan without much success.
- Camaïeu was placed in receivership in May 2020. In August, the Court of Lille authorized the takeover project carried out by FIB (Foncière Immobilière Bordelaise), FIB plans to keep 511 of the 634 stores and 2659 of the 3146 employees of the group.
- La Halle, an active brand in both clothing and footwear, which employs more than 6000 people in France and remains the property of Vivarte, has been on the bankruptcy watchlist since April.
- Damart, a clothing brand specializing in warm and comfortable under garments (head office in Lille, Nord), is cutting its workforce by 10% in order to restore profitability.

The sector will emerge from the crisis weakened. Mid-range brands have experienced the most difficulty during COVID-19. This crisis has revealed structural fragilities in terms of margin in a society with a shrinking middle class. Luxury brands have also suffered from the absence of foreign consumers during the summer holidays. Only smaller brands with loyal customers and shops providing personal touch services such as makeovers, have succeeded in overcoming the crisis.

### Behind the masks: ethics and corporate responsibility during the COVID-19 crisis. Case study

Mask production involves major issues like innovation, ethics and fashion. It can be considered as a case study useful in the analysis of textile challenges.

During lockdown, roughly 400 French companies manufactured masks. This production made it possible for the fashion and textile disaster-stricken sector to maintain minimal activity. Mask-making undertaken by numerous players in the industry has brought to light several revelations concerning their corporate responsibility and attitude to innovation. Some companies have produced masks, others have even gone as far as marketing them.

French luxury groups such as LVMH, Kering, Chanel and Hermès were active during the COVID-19 outbreak to help battle the spread of the virus. They announced that they would continue to pay their employees’ wages during the pandemic to spare the cost to the nation. In addition they also stated that they would grant financial aid to hospitals and launch clothing workshops to aid in the production of masks and gowns.

These groups decided not to take advantage of government aid payments and instead pick up the tab themselves. According to Chanel’s end of March official press release: “The goal is to not put pressure on the public purse, so that the French State can focus on helping more vulnerable businesses”. To further help combat the virus, Louis Vuitton, Christian Dior Couture, Loewe, Celine and Kenzo have all either been producing large quantities of alternative non-surgical face masks and hospital gowns or supporting their production and subsequent distribution.

With hospitals around the world facing shortages of materials and equipment, the private sector has quickly stepped up in the fight against COVID-19. Teams from both Louis Vuitton and Christian Dior have been working away for several weeks now after repurposing their production workshops in order to make non-surgical face masks for the general public. Christian Dior reopened its Redon workshop (IIle-et-Vilaine), which normally is purposed with producing their line of Baby Dior clothes. Volunteer workers produce personal protective equipment that is imperative to halting the transmission of the virus. Masks are distributed first to workers in contact with the general public in order to keep the country’s essential services running, these include supermarket cashiers, retailers and employees in government services. Christian Dior Couture is extremely proud of the strong sense of solidarity among its teams, without whom this initiative would never have been possible.

Louis Vuitton has geared up several of its French workshops to make large quantities of officially certified non-surgical face masks. Over 300 leatherworkers have been mobilized at twelve Louis Vuitton workshops around France: Marsaz and Saint-Donat (Drôme), Condé (Indre), Saint-Pourçain (Allier), Ducey (Manche) and Sainte-Florence (Vendée). The masks are being made in partnership with “Mode Grand Ouest”, a grouping of regional textile businesses, that is providing one of the key materials for production. These masks are also being distributed to the country’s most vulnerable, including, for example, the Marpa nursing home for seniors. In addition, Louis Vuitton is making gowns for frontline hospital workers in Paris, who are facing a serious shortage of personal protective clothing. The gowns are being made by volunteers at a workshop near Louis Vuitton headquarters in Paris.

Luxury groups have been exemplary in terms of corporate responsibility during the crisis.

During the crisis, certain lesser known manufacturers also decided to enter the face mask market. For example, Innovatex (Vendée), specialized in fabricating windsocks primarily used at airports to show direction and strength of wind, decided to create a mask designed for health professionals. They advocate the advantages of a mask that doesn’t fog up glasses and "keeps the nose and surrounding areas dry". Their masks feature an interior lining that drains water vapor and are made from 100% polyester that’s capable of withstanding high temperatures. The local designer opted for sublimation rather than print to avoid clogging the material with ink deposits therefore inhibiting its filtering ability. The price is set at 13€. Chantelle in Cachan (Paris) decided to reorganize their workshops in order to produce masks (category 1 and 2, reusable, for non-sanitary use). They meet AFNOR manufacturing standards and have been validated by the French Defense Procurement Agency. Each week, 500,000 masks are fabricated in their factories for the French group which currently has five separate brands: Chantelle, Passionata, Chantal Thomass, Femilet and Livera.

Mask-making appears also as a crucible for innovation in: lightness, technical fabrics, advancements in antiviral protection, improvements in breathing conditions… We can quote Géochanvre (Vosges): “we want to develop a mask made from natural hemp fibers that’s vegetable-based, organic and compostable”. Certain companies offer masks with a transparent view of the mouth as a solution for the deaf. In the Rhône region, Trajet-Aunde has developed fabrics that have the ability to self-disinfect without the need for a disinfectant. To achieve such a result, the textiles undergo a treatment based on titanium dioxide. In Isère, Serge Ferrari company invented a fabric with silver particles able to destroyed 99.5% of coronavirus in 1 h.

In the May 2020 edition of Vogue magazine, an article titled “Cloth Masks Available for Purchase” was published. Experts and trend forecasters are increasingly suggesting that masks may need to be worn for at least a year, until a vaccine is developed. During the spring of 2020, reports of new companies that were manufacturing masks or pivoting to offer them were frequent. During summer when mask wearing became compulsory in enclosed spaces (and some streets), masks became trendy and grew to be a part of daily life, donned by all with the same air of unconscious acceptance like a coat or sunglasses when leaving the house.

Depending on company values and beliefs regarding corporate responsibility, some businesses have decided to get involved in the mask-making trade and some have not. Before the coronavirus pandemic even began, masks had already been introduced into fashion collections. For example, those conceived by Marc Jacobs and Richard Prince for Louis Vuitton, those of Rick Owens and Alexander Wang, are all completely out of stock (now being sold for 3 times the price on resale websites). With exception to rappers, celebrities have not hesitated to don their masks on the red carpet, as seen by high profile stars such as Billie Eilish, Miley Cyrus and Ariana Grande.

Due to COVID-19, masks and fashion have come together again, but not all for the same reasons. While several brands have helped to fight against the coronavirus, either by making masks for caregivers, or by passing on their profits to hospitals in France, now more and more designers are embarking on the creation of fashionable masks. The principal is to help people find masks, protect themselves from the virus, while bringing themselves a little cheerfulness. So, is the mask a new fashion accessory to shop for until the end of lockdown? Cheaper than those of the brand Off-White, there are, for example, those of Pierre Talamon, a tailor in Paris selling them for €15 each. He thinks that masks are becoming a fashion accessory.

On the large luxury house side, there is complete silence on the subject of masks in fashion. Those who before lockdown had lent themselves to the trend of masks seem no longer to appreciate this ‘accessory’ as much, its connotation having changed since the pandemic. From their perspective now it would be inappropriate and vulgar to promote masks as fashion accessories. The mask is an object designed solely for one’s safety, one that shouldn’t be fooled with or invite delusions of grandeur.

## Discussion and evaluation

We focus on quality improvement and labelling as a strategy for French textiles in the present global context.

French textile firms should continue to promote innovation and upgrade quality in line with international trade model recommendations (Krugman, 1979). Incorporating increasing returns and love of variety, the Krugman model predicts that rich countries will have produced and exported a wide range of goods. France’s textile industry must continue to build on quality while striving to occupy high-end segments and market niches in the global market.

### Preliminary comments on textile quality

Quality continues to be crucial in turbulent modern global market. As a concept, quality proves to be difficult to define. For the principal cloths (raw silk, raw cotton, raw wool, etc.), a higher quality product makes sense, as there is a clear link between quality and the intrinsic properties of the raw materials themselves. For intermediary components, such as thread, reliability is crucial to quality: quality control aims to prevent faults. Ultimately, the final product’s perceived quality is subjectively created in the consumer’s mind. In textiles, a luxury article retains the same exceptional characteristics over a long period of time. Generally, luxury brands are founded on their high quality, uniqueness and exclusivity. The essence of luxury is also based on such deeper values as heritage, authenticity, and more recently, sustainability (Hennings et al., 2013; Joy et al., 2014).

Corporate social responsibility has become an integral part of strategy for global businesses. In the luxury textile trade, firms need to be truly exemplary. A culture that fully embraces ethical practices is expected of firms that represent high quality and excellence. Recent research also links luxury brands with social and environmental responsibility. A recent paper showed that luxury products, when built on quality and craftsmanship, can provide a solid basis for environmentally responsible communication (Nash et al., 2016). Properly formulated environmental messages do not degrade the constituents of luxury items, such as quality and uniqueness but rather enhance consumer perceptions of the luxury items’ worth. Considering prices and margins, the global consumer cannot accept a luxury product that is not in line with contemporary social values. CSR in the luxury textile industry often translates to working conditions for employees. Providing high quality working conditions is not only ethical but also key to achieving the levels of perfection consumers have come to expect in luxury goods. Even the largest levels of production remain within the limits of human hands. The preservation of know-how from certain rare suppliers is also a crucial matter. A lot of these rare craftsmen (feather workers, wooden hat block workers, lace makers, etc.) gravitate towards the ‘Grandes Maisons’. Haute Couture should value their diverse range of specialist suppliers that have played an important role in human history.

### Made in France and other quality-based labelling: success and setbacks

In today’s modern global world, a lot of textile companies have implemented French origin labelling strategies with mixed success. Made in France can’t be the one single argument for success, a distinctive well-rounded approach should include other more profound values such as: fair trade, being environmentally friendly or innovative. In order to succeed, the ‘Made in France’ strategy needs to be associated with quality-based classifications such as the EPV “Entreprise du Patrimoine Vivant” label (Living Heritage Company).

For non-food products, indication of geographical origin is optional (Blancheton & Hlady-Rispal, 2020). The use of this commercial argument is subject to compliance with certain rules so as not to cause confusion for the consumer. Indeed, in the context of evolving value chains with multi-country production, the origin of a product can be difficult to determine. Origin marking is based on customs rules, called "non-preferential rules of origin", defined in European regulations. They make it possible to establish the "nationality" of a product despite the fact that various countries contributed to its creation. These rules vary by the very nature of the finished product.

Simple finishes, handling and packaging are not enough to determine origin on a product. For products that have been assembled or processed in at least 2 countries, only the following are eligible for the "Made in France" label:

- products whose last substantial transformation or modification, having resulted in the creation of a new product, was carried out in France,
- products for which 45% of the value added was produced in France.

On this basis, a shirt made in France from Chinese fabric can be certified of French origin. The origin marking is done on a case-by-case basis for each product category. The criterion of "last substantial transformation" is generally expressed in one of the following 3 ways:

- by a change of heading or subheading in the customs tariff classification.
- by a list of processing operations which records the country origin in which these operations were carried out,
- by a proportion of value added in the ex-works (EXW) price of the products.

In order to promote stricter criteria, the Origin France Guaranteed label was launched in 2010 following a parliamentary report submitted to Nicolas Sarkozy. In the process, the association Pro France was founded in May 2010 by Yves Jégo. This label took the form of an association as European laws do not allow member nations to directly promote their own production. The principle labeling criterion states that at least 50% of the unit cost price must come from France. An independent audit then certifies the French origin of the products. Each company is thus granted a certification number and anyone, via an iOS or Android app, can check whether a particular product or company is of French origin or not.

The EPV label, created in 2005 by the French government, is a nationally recognized labelling system that distinguishes French companies for their excellent craftsmanship and industrial expertise. The label guarantees quality and confirms heritage and is well-recognized, which can bring national or even international media coverage to your activity, promote its development and is accompanied by an advantageous tax relief scheme (Blancheton, 2021). Eligibility is open to all forms of production, transformation, repair and restoration. It is awarded for 5 years after a rigorous candidate selection process. The following industries are considered eligible:

- Professions linked to art and culture, through their advanced working methods, the scarcity of their equipment and their cultural scope. Baccarat, Chaumet and Hermès are among such companies.
- Gastronomic professions, for the recognition of terroirs, specialties and prestigious haute cuisine such as Dalloyau, Fossier Biscuits, Puyricard Chocolate Factory, Petrossian Caviar and Bollinger Champagne.
- Industries which develop and use cutting-edge technologies, possess intellectual property with very high added value and safeguard traditional manufacturing FG certified.

In 2007, Thomas Huriez opened Modetic, an organic cotton clothes retailer in Romans-sur-Isère (Drôme), founded on environmental sustainability principles. In 2013, by the way of crowdfunding, he created the jeans brand “Jeans 1083” (1083 signifying the number of kilometers between the two most geographically separated towns of France, Menton and Porspoder). Since then, he has experienced considerable success, selling more than 100,000 organic cotton jeans. Mr Huriez is also attempting to manufacture his own French made textile, as evidenced by his purchase of one of the last standing cotton mills in France, located in Rupt-sur-Moselle (Vosges). Excluding buttons, rivets and the organic cotton he imports from Tanzania, Modetic’s entire value chain is located in France.

Atelier Tuffery has produced jeans and denim in France since 1892, originally in Florac and now situated in Saint-Julien-du-Gourg (Lozère). Devastated by the decline of the French textile industry, the company only had 2 workers at the start of the 2000s and produced a mere 300 jeans per year. Now, with EPV label accreditation, it has promoted Made in France and utilized French suppliers and digital marketing as its strategy to great effect. It now employs 15 people, produces 12,000 jeans per year (organic cotton and natural indigo) and generated € 1.2 million in turn-over in 2018.

The Blanc des Vosges textile house, created in 1843 in Vosges (an area with a rich textile history), employs over 100 people, produce high quality while trying to mix art and technology.

French textile companies that implement a Made in France marketing strategy are having difficulties. In 2017, the Limoges Commercial Court announced a 6 month renewable period of receivership for Smuggler, a French suit and menswear retailer. In March 2020, France Confection, the last manufacturer of French made suits and trousers, was placed in compulsory liquidation by the Limoges Commercial Court, leaving 85 employees without jobs.

Armor-Lux has become synonymous with Made in France ever since the French Ministry of Productive Recovery decided to promote the “la marinière” navy style attire of Armor-Lux in 2012. The buzz generated was extremely successful in increasing sales of the 1938 established Finistère-based company. But recent success should not hide the fact that only fifty percent of Armor-lux’s production is carried out in France.

Saint James (created in Manche, 1889) has roughly the same strategy with ‘marinière’ (horizontal blue and white striped sailor style shirts, pullovers etc.) sea lifestyle and woolen clothing. The company was bought by its own employees in the 1990s; it received EPV accreditation in 2013 and now exports 40% of its production.

The emergence of a high quality (and luxury) positioning strategy requires the mobilization of the company’s historical heritage. It does not necessarily reside in continuing an old-fashioned family inherited trade (skills, specific production techniques, etc.), but can simply evoke its celebrated past. Thus, as Carlo Belfanti recounts at the end of the nineteenth century, the emerging Lombard fashion referred to the clothing history and Italian renaissance colors (Belfanti, 2021). It reuses historical fabrics like silk velvets and especially lace, a fashion accessory much appreciated by Haute Couture who sometimes look for period copies. References to the Renaissance also concern the reminiscence of painters and colors like “Titian red” or “Veronese green”. France has a credible history in raising up new luxury centers in textiles based on their many historical regional traditions.

In South West France certain local companies (Moutet, Lartigue, Artiga, tissage de Luz, etc.) continue to produce ‘Basque linen’ for household use (tablecloth, towel and misc. accessories) in reference to deeper regional heritage. Originally, Basque linen fabric was made up of 7 colored stripes. It was used to protect agricultural livestock (oxen formerly present in the Basque region), against heat and parasites. On the traditional Basque linen, we still find the seven colored stripes. They represent the seven Basque provinces, three on the French side (Labourd, Basse Navarre and Soule) and four on the Spanish side (Avala, Navarre, Biscaye and Guipuzcoa). The colors made it possible to mark membership in a trade: blue for fishermen, green for farmers or red for breeders. The twisted cotton thread quality design of local companies faces the competition from cheap Asian imitations. These businesses appear to have the potential to develop and export their unique story-telling Basque linen.

Surf fashion in South West France has always tried to push a high-quality local product (wetsuits, swimwear, sweatshirts and others accessories), centered around their rich history in pioneering the sport in France at the end of the 1950s.

## Conclusions

This paper offers a global perspective of the textile and fashion industry in France during COVID-19 pandemic. This crisis is part of the long, slow decline that has already been taking place for several decades. In 2018, textiles represented only 2% of added value in the French manufacturing industry. During the last twenty-five years, it has lost two thirds of its workforce and production has been more than halved. This article shows that production levels in May of 2020, reached an all-time low 20 to 30% of that normally recorded. In retail, well known brands are facing extremely hostile economic times (Naf-Naf, La Halle, Catimini, Chipie, Camaïeu, Célio). COVID-19 has been a catalyst in the weakening of the sector as consumption continues to decline. The retail atomistic market structure is characterized by increasingly compressed margins. Many well-known brands have asked to have judicial safeguard procedures initiated.

Even if the shortage of sanitary masks has shown the virtues of local production and the sector’s ability to innovate (technical fabrics, advancements in antiviral fabrics), reshoring textile production back to France does not appear to be a viable solution. National manufacturers should continue to promote innovation and quality to survive in the present turbulent global environment. This paper reminds us that quality is a perception issue and that promoting labels on world markets can help the sector to ensure that quality perception. Developing clusters can also be a feasible solution particularly for high-end products in order to improve textile and fashion product visibility. France should capitalize on its strong credibility by expanding its luxury fashion and textile supply.

## Data Availability

Not applicable.
